# Serological and molecular tools to diagnose visceral leishmaniasis: 2-years’ experience of a single center in Northern Italy

**DOI:** 10.1371/journal.pone.0183699

**Published:** 2017-08-23

**Authors:** Stefania Varani, Margherita Ortalli, Luciano Attard, Elisa Vanino, Paolo Gaibani, Caterina Vocale, Giada Rossini, Roberto Cagarelli, Anna Pierro, Patrizia Billi, Antonio Mastroianni, Simona Di Cesare, Mauro Codeluppi, Erica Franceschini, Fraia Melchionda, Marina Gramiccia, Aldo Scalone, Giovanna A. Gentilomi, Maria P. Landini

**Affiliations:** 1 Unit of Clinical Microbiology, Regional Reference Centre for Microbiological Emergencies (CRREM), St. Orsola-Malpighi University Hospital, Bologna, Italy; 2 Department of Experimental, Diagnostic and Specialty Medicine, University of Bologna, Bologna, Italy; 3 Infectious Diseases Unit, Department of Medical and Surgical Sciences, University of Bologna, Bologna, Italy; 4 Public Health Authority Emilia Romagna, Bologna, Italy; 5 Unit of Microbiology, The Romagna Hub Laboratory, Pievesestina (Fc), Italy; 6 Infectious Diseases Unit, G.B. Morgagni Hospital, Forlì, Italy; 7 Infectious Diseases Unit, Azienda Ospedaliero-Universitaria Policlinico Modena, Modena, Italy; 8 Pediatric Hematology and Oncology Unit, St. Orsola-Malpighi University Hospital, Bologna, Italy; 9 Unit of Vector-Borne Diseases and International Health, Infectious Diseases Department, Istituto Superiore di Sanità, Rome, Italy; 10 Department of Pharmacy and Biotechnology, University of Bologna, Bologna, Italy; 11 Department of Research, Innovation and Technology, Istituto di Ricovero e Cura a carattere scientifico, Istituto Ortopedico Rizzoli, Bologna, Italy; Taibah University, SAUDI ARABIA

## Abstract

The diagnosis of visceral leishmaniasis (VL) remains challenging, due to the limited sensitivity of microscopy, the poor performance of serological methods in immunocompromised patients and the lack of standardization of molecular tests. The aim of this study was to implement a combined diagnostic workflow by integrating serological and molecular tests with standardized clinical criteria. Between July 2013 and June 2015, the proposed workflow was applied to specimens obtained from 94 in-patients with clinical suspicion of VL in the Emilia-Romagna region, Northern Italy. Serological tests and molecular techniques were employed. Twenty-one adult patients (22%) had a confirmed diagnosis of VL by clinical criteria, serology and/or real-time polymerase chain reaction; 4 of these patients were HIV-positive. Molecular tests exhibited higher sensitivity than serological tests for the diagnosis of VL. In our experience, the rK39 immunochromatographic test was insufficiently sensitive for use as a screening test for the diagnosis of VL caused by *L*. *infantum* in Italy. However, as molecular tests are yet not standardized, further studies are required to identify an optimal screening test for Mediterranean VL.

## Introduction

Visceral leishmaniasis (VL) is a serious disease caused by obligate intracellular protozoa belonging to the *Leishmania donovani complex* and associated with considerable morbidity and mortality. *Leishmania* is transmitted by the bite of sandflies of the genus *Phlebotomus* and *Lutzomyia* and is endemic in around 100 countries including Southern Europe, Asia, Africa, and Latin America [1, 2].

In Mediterranean Europe, VL is caused by *Leishmania infantum*, which is mainly transmitted by *Phlebotomus perniciosus*, *P*. *perfiliewi*, *P*. *ariasi*, *P*. *neglectus* and *P*. *tobbi* [3]. VL is endemic in South-Eastern and Western Europe, with about 500 new autochthonous cases reported annually [4]. The incidence of leishmaniasis has increased since the early 1990s; this phenomenon occurred particularly in Spain, France and Italy, the latter facing a spread in the North of the country [5–9]. Because of its high endemicity and the recent outbreaks in low endemic areas, leishmaniasis is becoming a public health concern in Mediterranean Europe and improved diagnostic methods are needed to identify this disease [10].

The diagnosis of VL was traditionally based on serologic testing and the direct demonstration of *Leishmania* by microscopic examination of bone marrow aspirates, but limits have to be considered, including low sensitivity of serologic testing in immunosuppressed patients [11], the necessity to use invasive procedures to obtain bone marrow samples, the low sensitivity of microscopy examination (between 53% and 85%) and the need of expert microscopists [12]. Over the past 10 years, molecular diagnostic tests have been developed, and polymerase chain reaction (PCR) targeting the *Leishmania* kinetoplast DNA or ribosomal RNA genes in clinical samples represents a highly sensitive technique for the diagnosis of VL [13, 14]. Nevertheless, there is a lack of standardization for PCR tests, and whether molecular methods should be considered the gold standard for diagnosis of VL has not yet been clarified.

Since the beginning of 2013, an increased number of leishmaniasis cases has been observed in the Emilia-Romagna region, Northeastern Italy [15], including an outbreak of VL in the Province of Bologna [8]. Following the increasing number of patients affected by leishmaniasis in this area, the Regional Reference Centre for Microbiological Emergencies (CRREM) in Bologna implemented an innovative diagnostic workflow for VL. In 2013, an integrated diagnostic strategy was employed, combining serological and molecular tests with standardized clinical criteria.

## Materials and methods

### Settings and samples

Human leishmaniasis is a compulsory notifiable disease in Italy [2]; laboratory confirmed cases of VL are reported by local public health departments to the Regional Authorities using a standardized notification form. During a 24-month period (from July 2013 to June 2015), data were collected from patients with suspected VL in several secondary care centers located in the Emilia-Romagna region (Italy), including the Provinces of Bologna, Modena, Parma, Reggio Emilia, Forlì-Cesena, Ravenna and Rimini; first line serological tests for VL were performed in different laboratories, including the Microbiology Units at the Romagna Hub Laboratory, Pievesestina (Forlì-Cesena) and the Policlinico Modena Hospital. Molecular tests and confirmatory serological tests were performed at the CRREM laboratory, Microbiology Unit, St. Orsola-Malpighi University Hospital, Bologna, Italy. A sample-accompanying form, including demographic details, travel history, clinical picture and laboratory data, HIV status or presence of other underlying immunosuppressive conditions, was filled in by the treating physician and sent to the CRREM laboratory. This is a retrospective observational study; data included in the study were collected from the database of the CRREM laboratory, Unit of Microbiology, Bologna. Participants written or verbal consent was not required for the following reasons; 1. the Microbiology Unit had no contact with the patients that referred to various secondary care centers of the Emilia-Romagna region (Italy), 2. the number of patients included in the study was high (ie 634 patients), 3. points 1 and 2 were among those admitted in the regulation issued by the Privacy Authority (General Authorization n. 9 about processing of personal data carried out for scientific research purposes, December 11, 2014). This study was submitted to the Ethical Committee of the St.Orsola-Malpighi University Hospital that approved the conduction of the research (prot. n.1049/2016).

### Diagnosis of VL

Diagnosis of VL was based on the case definition of the World Health Organization, i.e. a patient showing characteristic VL clinical signs with serological and/or parasitological confirmation [1]. Clinical signs suggestive of VL included prolonged irregular fever, splenomegaly, hepatomegaly, loss of weight, while laboratory data included anemia, thrombocytopenia, leukopenia and hypergammaglobulinemia. The presence of a hemophagocytic syndrome was also considered as a suggestive sign of VL. An integrated diagnostic strategy for VL was applied; standardized clinical evidence was combined with frontline serological test supported by real-time PCR (RT-PCR) as the second line parasitological test. All patients in whom parasitological investigations tested negative for VL were followed up until another diagnosis was reached or symptom remission occurred. No patient was lost to follow up.

### Serological testing for VL

Serologic diagnosis was performed by rapid rK39-based immunochromatographic test (ICT; Rapydtest, Diagnostic International Distribution S.p.A, Milan, Italy) that qualitatively detects antibodies that are specific for the recombinant *Leishmania* antigen rK39 [16]. IgG anti-*L*. *infantum* antibody titer was measured by indirect immunofluorescence assay test (IFAT; bioMerieux, Marcy l’Etoile, France). According to the manufacturer’s instructions, sera were considered positive when specific IgG titer was equal to or above 1/160.

### Molecular testing for VL

Home-made molecular methods were used to detect leishmanial DNA in bone marrow aspirate and/or peripheral blood specimens. Nucleic acids were extracted from 100 μL buffy coat (peripheral blood samples), 100 μL whole blood (peripheral blood) and/or 100 μL bone marrow aspirates with NucliSENSeasyMAG (Biomerieux, Marcy l’Etoile, France). DNA was eluted in 50μL of elution buffer. DNA was amplified employing simultaneously two RT- PCR assays, by the amplification of 1. a segment of the small-subunit ribosomal (r)RNA gene [17] and 2. a segment of the leishmanial kinetoplast (k)DNA [18]. Primers (U1 5’-AAGTGCTTTCCCATCGCAACT-3’, U2 5’-GACGCACTAAACCCCTCCAA-3’ for rRNA PCR; 15 pmol of RV1 5’-CTTTTCTGGTCCTCCGGGTAGG-3’, 15 pmol of RV2 5’-CCACCCGGCCCTATTTTACACCAA-3’ for kDNA PCR) were synthesized by PrimmBiotech (Milan, Italy) and 50 pmol of TaqManprobes (FAM-CGGTTCGGTGTGTGGCGCC-TAMRA and FAM-TTTTCGCAGAACGCCCCTACCCGC-TAMRA for rRNA PCR and kDNA PCR, respectively) were synthesized by IDTDNA (Leuven, Belgium). The RT-PCR assays were performed by employing the CFX Real Time PCR detection System (Bio-Rad, California, USA). β2-microglobulin RT-PCR assay was run simultaneously as a control of amplification of the extracted DNA.

### Diagnostic workflow for VL

**Fig 1:**
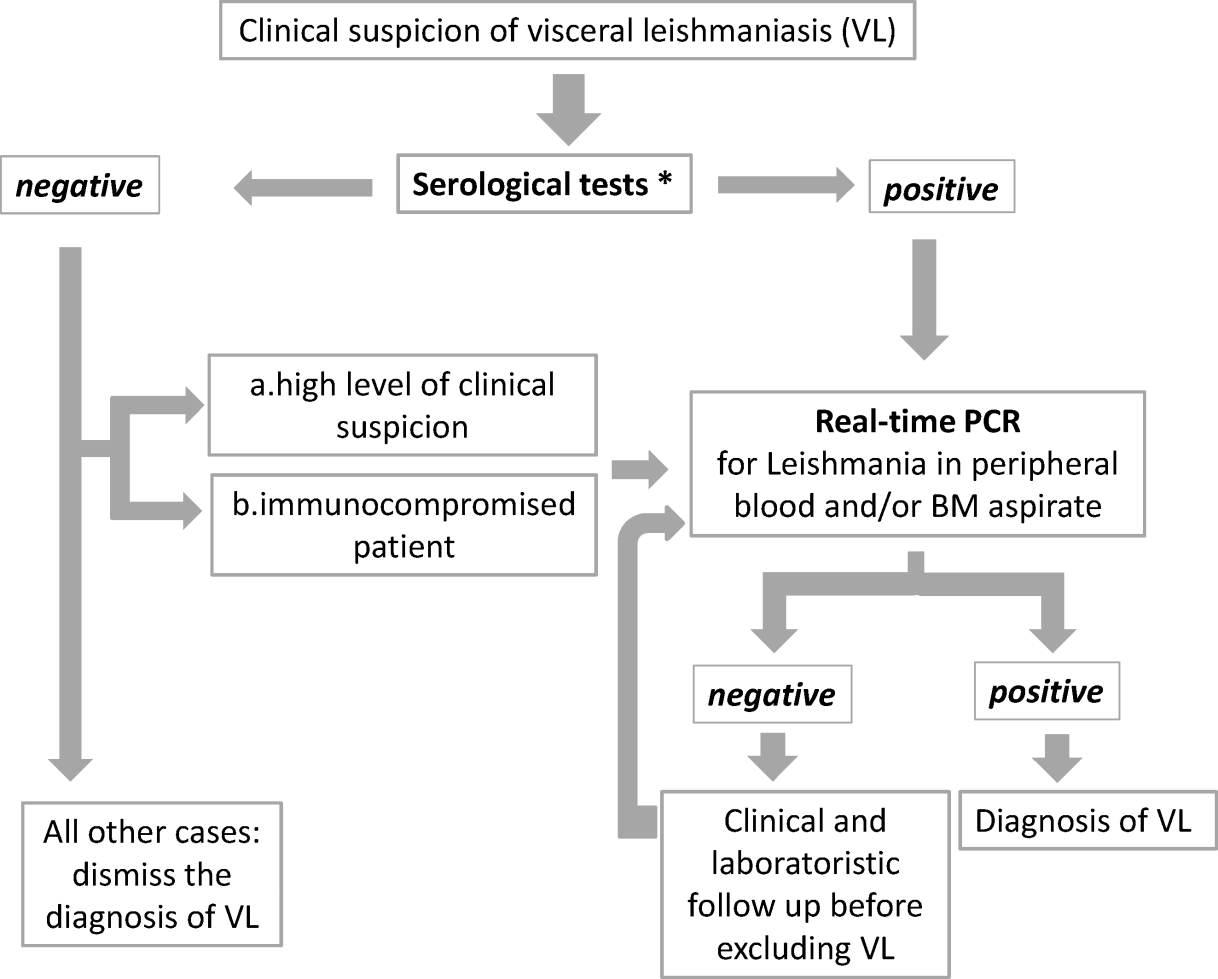
Diagnostic workflow for visceral leishmaniasis (VL). *As serological tests, the rapid rK39-based immunochromatographic test (ICT) was performed. Subsequently, the indirect immunofluorescence test (IFAT) was carried out for antibody titration in all ICT-positive cases. IFAT was also performed in PCR-positive/ ICT-negative cases and when test result for ICT was uncertain. BM, bone marrow.

A diagnostic protocol was implemented in all cases of suspected VL during a 24-month period (Fig 1). First, rapid rK39-based ICT was performed as a screening test in all sera from individuals with suggestive clinical signs and laboratory data. In all ICT-positive cases, RT-PCR tests were carried out. Positivity of serology and/or RT-PCR in patients with suggestive clinical picture and laboratory data were considered confirmatory for VL diagnosis.

To combine serology results with clinical data and to perform a strict monitoring of suspected VL cases, clinical and laboratory staff of secondary and tertiary care centers in the Emilia-Romagna region was involved; diagnosis of VL was dismissed in rK39 ICT-negative cases when spontaneous resolution of symptoms was achieved or another diagnosis was obtained. Conversely, if the patient was immunocompromised and/or the clinical suspicion persisted, and/or no other diagnosis was identified, molecular assays were performed to detect leishmanial DNA, regardless of the rK39 ICT results. As confirmatory assay, IFAT was performed in the following cases; 1. in all ICT-positive cases, for antibody titration; 2. in RT-PCR-positive/ICT-negative cases; 3. when the test result for ICT was uncertain. Finally, close clinical follow up of patients with suspected VL was performed and additional peripheral blood and/or bone marrow aspirates were sent to the CRREM laboratory whenever worsening of clinical condition was observed in the absence of alternative diagnosis.

### Statistical analysis

Descriptive statistics were used to summarize demographic and clinical characteristics. By means of true positive, true negative, false positive and false negative rates we computed sensitivity and specificity. Statistical analysis was performed by using SPSS v. 20.0 (IBM Corp., Armonk, NY, USA)

## Results

In the study period, 634 serum specimens were collected from an equal number of patients with clinical signs and laboratory data that were suggestive for VL. Sera were tested by the rK39 ICT. VL diagnosis was dismissed in 540 cases due to a combination of ICT-negative results and a low level of VL suspicion, spontaneous resolution of symptoms, or when another diagnosis was reached. Conversely, molecular tests were subsequently performed in specimens from 94 patients with ICT positive result and/or a high level of clinical suspicion and/or presence of immunosuppression. In 32 out of 94 cases, bone marrow aspirate was available for RT-PCR testing.

The presenting clinical features of these 94 patients were prolonged/irregular fever (74 out of 94; 78.7%), splenomegaly (52 out of 94; 55.3%), hepatomegaly (32 out of 94; 34%), lymphadenopathy (22 out of 94; 23.4%), and weight loss (27 out of 94; 28.7%), while the main laboratory findings were anemia (58 out of 94; 61.7%), thrombocytopenia (51 out of 94; 52.2%), leukopenia (49 out of 94; 52.1%), and hypergammaglobulinemia (14 out of 94; 14.9%). Sixty-one patients (65%) were males; the median age was 49.5 years (range 1 month– 86 years). Twelve patients (13%) were children (range 1 month– 11 years), and 15 patients (15.9%) were HIV-positive. Based on serological, molecular and clinical criteria, 21 out of 94 (22.3%) patients were confirmed as VL cases, including 6 immunocompromised patients; 4 of these patients had HIV infection, one patient suffered from advanced-stage lymphoma and one patient was under cyclosporine and steroid treatment because of myasthenia gravis (Table 1). The age of the *Leishmania*-infected patients ranged from 24 to 86 years, with a median age of 57. All VL cases were adult, with only one patient in our study group under 30 years of age (24 years). The male-to-female ratio was 19/2. Thirteen out of 21 patients were resident in the Bologna Province, while the other 8 were resident in Modena (n = 2), Forlì-Cesena (n = 2), Ravenna, Rimini, Parma, and Reggio Emilia Provinces, respectively.

**Table 1:** Clinical data, laboratory tests and outcome of patients with visceral leishmaniasis, July 2013 –June 2015.

		Total patients n = 21	
	Nr.		%
**Clinical characteristics of VL**			
Fever	19		90.5
Splenomegaly	20		95.2
Hepatomegaly	10		47.6
Weight loss	11		52.4
Lymphadenopathy	7		33.5
**Co-morbidities**			
HIV	4		19.0
Alcoholism	3		14.3
Advanced-stage lymphoma	1		4.7
Obesity	1		4.7
Pulmonary sarcoidosis	1		4.7
Pulmonary thromboembolism	1		4.7
Mycosis fungoides	1		4.7
Myasthenia gravis	1		4.7
**Laboratory tests**			
Anemia	19		90.5
Leukopenia	18		85.7
Thrombocytopenia	20		95.2
Hypergammaglobulinemia	8		38.0
**Outcome°**			
Complete resolution	11		61.1
Death	3*		16.6
Relapses or chronic active infection in HIV	4		22.2

VL, Visceral leishmaniasis

* 1 death for causes other than leishmaniasis

° Data not available for three patients

All 73 patients that tested negative for VL by serological and molecular tests were followed up until another diagnosis was reached or up to clinical remission. No patient was lost to follow up.

The performance of serological tests and RT-PCR assays was evaluated (Table 2). As a screening test, rK39 ICT was performed in sera from 92 out of 94 patients; 2 sera were not available for analysis. In ten out of 21 VL cases, rK39 ICT gave negative results, including 3 HIV-positive patients, with an overall sensitivity of 52.4%. By restricting the results to immunocompetent patients (n = 15), the sensitivity and specificity of rK39 ICT were 60% and 96.5%, respectively. When evaluating sera from 71 VL-negative patients, the rK39 ICT tested positive in 3 cases (Table 2); all three cases tested negative with RT-PCR. The first case was an HIV-positive patient with previous VL (diagnosed and treated 18 months earlier) and currently presenting with mild, self-limiting fever. Because of the self-resolving fever and the lack of other clinical and laboratory signs of VL, this case was considered as a HIV/*Leishmania* co-infection case with no active *Leishmania* infection and was not included among the VL cases. In the two remaining cases there was no previous history of VL; these patients presented with fever; in one case, fever and cutaneous rash resolved spontaneously within one week, while the other case was diagnosed as a T-cell lymphoma. On the basis of the clinical follow up of these two patients, VL diagnosis was ruled out, despite testing positive at serology.

**Table 2:** Performance of serological and molecular tests for diagnosis of visceral leishmaniasis, VL (n = 94). VL cases were identified by comprehensive diagnostic criteria, ie clinical and parasitological criteria.

	VL-positive cases	VL-negative cases	Sensitivity (95% CI)	Specificity (95% CI)
**rK39 ICT+**	11/21	3/71°	0.52 (0.31–0.74)	0.96 (0.91–1.00)
**IFAT+**	18/20	2/19	0.90 (0.77–1.03)	0.89 (0.76–1.03)
**Real-time PCR+**	21/21*	0/73	1.00 (1.00–1.00)	1.00 (1.00–1.00)

VL; Visceral leishmaniasis, CI; confidence interval, ICT; immunochromatographic test, IFAT; immunofluorescence assay test, PCR; polymerase chain reaction

°2 sera were not available

*18/21 tested positive with both primer sets, rRNA and kDNA, 3/21 tested positive with primer set for kDNA and negative with primer set for rRNA.

Serum samples from 39 out of 94 patients were also tested by IFAT; among the VL cases, IFAT tested positive in 8 out of 10 rK39 ICT-negative cases; IFAT titers were low (1/160-1/320) in 5 out of these 8 cases. By pooling the results of rK39 ICT and IFAT, the sensitivity of serological tests in detecting VL cases was 85.7%.

Among the VL-negative patients, IFAT tested positive in 2 out of 19 cases; both cases also tested positive by rK39 ICT and were mentioned above as 1. an HIV-patient previously suffering from leishmaniasis and 2. a case of self-resolving febrile rash. As mentioned above, despite testing positive at serology, these patients were not considered as VL cases on the basis of molecular data and clinical follow up. IFAT tested positive in 18 out of 20 patients that were diagnosed with VL; two IFAT-negative results were obtained in HIV-positive patients suffering from VL. One patient’s serum was not available for IFAT. The sensitivity of IFAT was 90.0% and the specificity was 89.5%.

The case definition relied strongly on clinical data and follow up of the patients. Further, RT-PCR performed on peripheral blood and bone marrow aspirates exhibited equivalent sensitivity (data not shown).

## Discussion

Mediterranean VL usually affects children (1–4 years of age) or immunocompromised adults [1]; instead, 71% of the VL cases in our study cohort were adults with no clear signs of immunosuppression. In agreement with literature data [1], we observed that clinical disease was more frequent in men than in women, reflecting an increased exposure of men to sandflies and/or immunomodulation caused by androgens [19].

VL diagnosis was performed by a combination of clinical and parasitological data; the association of serological and molecular tools has become, in reference centers, a common approach to the differential diagnosis of VL, because of serious prognostic implications of an incorrect or late diagnosis of VL (as reviewed in [20]). Over the last decade, nucleic acid testing by PCR has emerged as a highly sensitive and specific diagnostic method to detect leishmanial DNA [13, 14, 20–24]. Various PCR assays have been validated for the diagnosis of VL, demonstrating higher sensitivity compared to microscopy [13, 22–24]. In addition, studies performed in dogs indicate that PCR can identify oligosymptomatic infections caused by *Leishmania* [25–27]; similarly molecular tools may contribute to detect *Leishmania* infection in patients presenting with atypical symptoms or with paucisymptomatic infection.

A considerable amount of evidence indicates that ICT based on rK39 − a kinesin-related protein of parasites belonging to the *Leishmania donovani* complex − is a sensitive and specific method for the serological diagnosis of VL in patients with febrile splenomegaly and no previous history of the disease in the Indian subcontinent [16, 28], while a lower sensitivity of rK39 ICT has been reported in east Africa [29, 30]. Studies reporting the diagnostic accuracy of rK39 ICT in samples from VL-patients in Mediterranean Europe indicate optimal performance of rK39 ICT in Southern Italy [31] and Spain [32], while other rapid tests, such as rKE16 ICT exhibit lower sensitivity in VL samples from France [33]. As we mainly expected autochthonous cases of VL, we chose to employ a rK39 ICT as the frontline test in our diagnostic workflow. Unexpectedly, we observed a suboptimal sensitivity of the rK39-based test in both immunocompetent and immunocompromised patients. Negative results from the rK39 ICT in VL cases were associated with positive IFAT in 8 out of 10 cases, suggesting that false negative results were obtained by rK39 ICT.

The reason for the low sensitivity of the rK39 ICT in our patient cohort is unclear and may reflect the genetic diversity of the rK39 homologous sequences in different *Leishmania* strains [29, 34]. However, we cannot exclude the possibility that our results may be related to a varying quality of the antigen preparation for setting up the rK39 ICT.

By pooling together rK39 ICT and IFAT results, the sensitivity of serological tests reached 85.7% for VL diagnosis; IFAT proved to be better than rK39 ICT and may be considered as a good option for VL screening if molecular tests are not standardized. Nevertheless, we did not employ IFAT as frontline test; thus, additional studies comparing the clinical utility of IFAT as screening tests for VL in Northern Italy are warranted to confirm this matter.

One of the major drawbacks of serological tests is the fact that they cannot be used to detect relapses because the antibody remains present long after clinical cure [35]; in our cohort, one HIV-positive patient with previous VL tested positive by rK39 ICT and by IFAT. Nevertheless, the lack of detection of parasitic DNA in peripheral blood contributed, together with clinical data, in identifying this case as a past leishmanial infection.

The main limitation of our study is that patients with clinical and laboratory data suggestive of VL entered the diagnostic algorithm based on clinical signs and rK39 ICT results. As the rk39 ICT subsequently showed low sensitivity, we cannot rule out the possibility that ICT-negative VL cases could have been excluded at an earlier stage of infection (low clinical suspicion) from the diagnostic workflow and subsequently lost to follow-up. VL is a compulsory notifiable disease in Italy [2] and VL-confirmed cases are obligatorily reported by local public health departments to the regional health authorities, which monitor cases of human leishmaniasis. As no VL cases were reported to the Regional Health Authority among the ICT-negative patients with low level of clinical suspicion that were disregarded by our study, we assume that no VL cases were excluded from the study because of the modest performance of the rk39 ICT.

In conclusion, we employed an integrated diagnostic approach for Mediterranean VL, which combined standardized clinical evidence and parasitological diagnosis by molecular and serological tests. Based on our experience, the rK39 ICT is insufficiently sensitive to be used as a screening test for diagnosis of VL caused by *L*. *infantum* in Italy. The inclusion of molecular tests in all cases of high level clinical suspicion and in immunosuppressed patients made it possible to detect all VL cases that were notified to the Regional Health Authority. However, as molecular tests are yet not standardized, further studies are required to identify an optimal screening test for Mediterranean VL.

## Abbreviations

CRREM: Regional Reference Center for Microbiological Emergencies
ICT: immunochromatographic test
IFAT: indirect immunofluorescence assay test
kDNA: kinetoplast DNA
RT-PCR: real-time polymerase chain reaction
rRNA: ribosomal RNA
VL: visceral leishmaniasis
